# High prevalence and risk factors of dropout intention among Chinese medical postgraduates

**DOI:** 10.1080/10872981.2022.2058866

**Published:** 2022-03-31

**Authors:** Pu Peng, Winson Fuzun Yang, Yueheng Liu, Shubao Chen, Yunfei Wang, Qian Yang, Xin Wang, Manyun Li, Yingying Wang, Yuzhu Hao, Li He, Qianjin Wang, Junhong Zhang, Yuejiao Ma, Haoyu He, Yanan Zhou, Jiang Long, Chang Qi, Yi-Yuan Tang, Yanhui Liao, Jinsong Tang, Qiuxia Wu, Tieqiao Liu

**Affiliations:** a National Clinical Research Center for Mental Disorders, and Department of Psychiatry, The Second Xiangya Hospital of Central South University, Hunan, Changsha, China; b Department of Psychological Sciences, Texas Tech University, Lubbock, Tx, USA; c Department of Psycholoy, College of Education, Hunan First Normol University, Changsha, China; d Department of Psychiatry, Hunan Brain Hospital (Hunan Second People’s Hospital, Changsha, China; e Institute of Mental Health Management (SJTU/CHDI), Shanghai Mental Health Center, Shanghai Jiao Tong University School of Medicine, Shanghai, China; f Department of Psychiatry, Zhejiang Provincial People’s Hospital, People’s Hospital of Hangzhou Medical College Hangzhou, . Zhejiang, P.R China; g College of Health Solutions, Arizona State University, Phoenix, AZ, USA; h Department of Psychiatry, Sir Run Run Shaw Hospital, School of Medicine, Zhejiang UniversityHangzhou, Zhejiang, P. R China

**Keywords:** Psychological distress, dropout, medical students, postgraduate medical education, career choice

## Abstract

**Background:**

A high attrition rate in medical students has exacerbated the physician shortage in China. However, few studies have explored the risk factors of dropout intention in medical postgraduates. This study compared the prevalence of dropout intention and mental distress between medical and non-medical postgraduates in China and investigated risk factors for dropout intention. This study also explored the impact of medical postgraduates’ perception of the Chinese healthcare environment on their mental status and dropout intention.

**Methods:**

This cross-sectional study was conducted using online questionnaires from October 2020 to April 2021. Convenience sampling was used to recruit postgraduates in different majors. Outcomes included dropout intention and potential risk factors, including mental distress, quality of life, and fatigue. Medical postgraduates were additionally assessed for healthcare environment satisfaction, burnout, career choice regret, and experiences of workplace violence. A logistic regression model was constructed to evaluate the association between dissatisfaction, mental distress, and turnover intention.

**Results:**

A total of 740 medical and 670 non-medical postgraduates participated in the survey. The rates of depression symptoms (33.8% vs. 39.0%, p < 0.001), anxiety symptoms (22.2% vs. 32.4%, p < 0.001), and somatic symptoms (34.7% vs. 42.4%, p = 0.004) were lower in medical postgraduates, while more medical postgraduates (58.4% vs. 48.4%, p < 0.001) reported dropout intention. Dissatisfaction with the healthcare environment (odds ratio [OR]: 1.65; 95% confidence interval [CI]: 1.17–2.34, p = 0.005), career choice regret (OR: 6.23; 95% CI: 4.42–8.78, p < 0.001), and high perceived stress (OR: 2.74; 95%CI: 1.90–3.94, p < 0.001) remained independently associated with turnover intention.

**Conclusions:**

Mental distress is common among postgraduates, calling for timely interventions. Medical postgraduates reported higher turnover intention. Healthcare environment perception also affected the mental health and dropout intentions of medical students. A decent future income, reduced workload, shorter duration medical training, and better doctor-patient relationships are urgently needed.

## Introduction

Despite different healthcare systems, doctor shortages are a worldwide problem severely affecting both patients and physicians [1–5]. High demands placed on the healthcare workforce, a large number of physicians leaving patient care, and a low rate of graduates entering the medical profession have exacerbated this issue at a global scale. The problem is even more severe in China, due to the huge population and poor doctor-patient relationships in the country [6]. China has only 0.14 general practitioners and 0.25 nurses per 1,000 people [6], and the country’s medical professionals suffer from decreasing public trust, a declining reputation, and adverse media reporting [7–9]. Violence and negative comments on healthcare providers have increased in China, which have been strongly associated with the decision to leave jobs in the sector [8,10–12]. Moreover, there has been a huge reduction in Chinese medical postgraduates deciding to enter clinical work, with only a small proportion of medical postgraduates (752,233 out of 4,314,791 students, 15.91%) registering as doctors in the past decade [13]. This sharp reduction may result from reduced attractiveness of the medicine profession, due to a decreasing pass rate for physician qualification examinations, high costs, a long training period, an extreme workload, violence and insults from patients, and lower income [6,14–16]. This situation was exacerbated by the emergence of the coronavirus disease 2019 (COVID-19) [17,18].

Postgraduates have become important to the future healthcare workforce since the reform of postgraduate medical education and standardized residency training [19–21]. According to the China Health Statistical Yearbook, the number of medical postgraduates reached 271,406 in 2018, nearly double that of 2008 [22]. The proportion of doctors with master’s or doctorate degrees in China increased from 11.4% in 2010 to 20.3% in 2018 [22]. Different from other countries, China has a three-tier medical education system, which includes undergraduate, master’s, and doctoral stages. In China, medical postgraduates are required to complete their academic research and clinical work at the same time, and they usually undergo more years of training than their peers in non-medical majors [23].

Previous studies found the dropout rate of medical students to range from 3.8% to 26% [24–31]. Some studies demonstrated a strong relationship between psychological problems (i.e., depression, burnout, anxiety, alcohol abuse) and the risk of dropout [30,32–34]. However, the majority of the investigations were conducted in countries other than China. To date, only a small number of published studies have focused on the risk of dropout in Chinese medical students. A cross-sectional study found that 6.9% of the 1,837 medical students reported decreased willingness to become a doctor after the outbreak of COVID-19, which was strongly associated with depressive symptoms, low professional satisfaction, young age, being female, low income, and negative news about the pandemic [35]. Another cross-sectional study revealed that nearly 60% of 2,922 medical undergraduates expressed turnover intention, which was attributed to the declining reputation of doctors, low social support, and low resilience [36]. However, those studies mainly focused on undergraduate medical students. Only one retrospective study assessed the incidence of dropout in postgraduate medical students and identified the reasons, which included poor academic performance and psychological problems [37]; nevertheless, it was published in Chinese, and few international readers had access to it. Furthermore, the above study was based on reports from only one local university. Therefore, the prevalence and correlation of dropout intention among Chinese medical postgraduates remained largely unknown.

To bridge this gap, this web-based cross-sectional study was conducted. The aim of the study was: 1) to assess and compare the prevalence of several mental distress symptoms (i.e., depression symptoms, anxiety symptoms, somatic symptoms, alcohol abuse/dependence, high perceived stress, and daytime sleepiness) and dropout intention between medical and non-medical postgraduates and 2) to investigate the possible association between mental health status, dissatisfaction with the healthcare environment, and dropout intention among medical postgraduates.

## Method

### Study design and participants

A cross-sectional online survey using structured questionnaires was conducted from 20 October 2020 to 5 April 2021. During this period, the COVID-19 pandemic had subsided in most areas of China and postgraduates had returned to campus or hospitals. The online questionnaire was distributed to postgraduates via an online survey platform (www.wjx.cn) and WeChat. Only participants who responded to all questions were able to submit the questionnaire. Participants gave their informed consent prior to starting the survey, and a report on their mental health status was presented immediately upon completion of the survey.

### Survey measures of main outcomes

The online survey contained three sections. Section 1 was related to the responder’s demographic characteristics, including age, gender, major, relationship status, degree applied for, and monthly income.

In Section 2, thoughts on dropping out were assessed using a two-item questionnaire from Dyrbye LN [30]. In this section, participants were asked whether they had thought about dropping out of school or changing to another medical or non-medical major within the last year. Participants who responded ‘yes’ were then asked about how strong these thoughts were. Alcohol abuse/dependence, depression symptoms, anxiety symptoms, daytime sleepiness, perceived stress, and somatization were assessed using the Chinese version of Alcohol Use Disorders Identification Test-Concise (AUDIT-C), the nine-item Patient Health Questionnaire (PHQ-9), the seven-item Generalized Anxiety Disorder Scale (GAD-7), the Epworth Sleepiness Scale (ESS), the 10-item Perceived Stress Scale (PSS), and the 15-item Patient Health Questionnaire (PHQ-15), respectively. All of the above scales had been validated and widely used among the Chinese population [38–41]. A cutoff point of 10 on PHQ-9, GAD-7, and PHQ15 was used to define moderate or severe depression symptoms, anxiety symptoms, and somatic symptoms. Females with an AUDIT-C score of ≥3 or males with a score of ≥4 were regarded as being positive for alcohol abuse/dependence. Cutoff points of 19 and 11 on PSS and ESS, respectively, were used to identify high perceived pressure and excessive daytime sleepiness. We adopted a standardized linear analog to assess the quality of life (QOL) and fatigue [42]. Participants with a score of ≥1/2 standard deviation (SD) below the gender-matched mean for the group were regarded as having a low QOL and having a high level of fatigue.

In Section 3, the medical students were assessed for burnout, career choice regret, experience of violence, and satisfaction with the medical environment. Burnout was assessed by two single items from Maslach Burnout Inventory (MBI) (i.e., ‘How often do you feel burned out from your medical learning?’ and ‘How often do you feel you’ve become more callous toward people since you entered the medical college?’). Students who reported emotional exhaustion or depersonalization at least weekly were considered as experiencing burnout. It was widely used by medical students and proved to be an abbreviated burnout assessment tool [43,44]. Participants were required to select the reason(s) for their regrets about studying medicine in a multiple-choice question. The options included ‘low income,’ ‘poor doctor-patient relationships,’ ‘long study period,’ ‘overwork,’ ‘intense competition,’ ‘experience of violence,’ ‘high expectations of patients,’ and ‘other causes.’ This question was obtained from the Chinese Physicians’ Practice Status White Paper released by the Chinese Medical Doctor Association [45].

## Statistical analysis

The normality of variables was tested using the Shapiro–Wilk normality test and the KS normality test. Continuous variables were presented as means ± SD for normally distributed data and medians and interquartile ranges (1st quartile, 3rd quartile) for non-normally distributed data. The chi-square and Wilcoxon rank-sum tests were used to compare inter-group differences in demographic data and mental health conditions as appropriate. Multivariate stepwise logistic regression was used to assess the association of violence, dissatisfaction, and career choice regret with dropout intention and several mental health problems, including depression symptoms, anxiety symptoms, somatic symptoms, alcohol abuse/dependence, high perceived stress, and daytime sleepiness. All analyses were performed using SPSS 26.0 (IBM Corp); all the tests were two-tailed, with p < 0.05 indicating statistical significance.

## Results

### Socio-demographic characteristics

A total of 1,613 postgraduates participated in this survey. Through screening for usable data, 203 respondents with logic errors in their responses (such as wrong answers for the Chinese national day) or studying overseas were excluded from the analysis. Thus, a total of 1,410 questionnaires were included in the final analysis; of these, 740 (52.5%) were from medical postgraduates. The median age of the entire sample was 24 (23, 26) years. Approximately 73.5% of the participants were female, and 28.5% were studying for a doctoral degree (Table 1).

**Table 1.** Demographic characteristics among medical postgraduates and non-medical postgraduates

Item	All (n = 1,410)^a^,	Medical postgraduates (n = 740)^a^	Non-medical postgraduates (n = 670)^a^	P-value^a^
Gender, n (%)				0.04
Male	374 (26.5)	179 (24.2)	195 (29.1)	
Female	1,036 (73.5)	56 1(75.8)	475 (70.9)	
Age, median (IQR)	24 (23,26)	25 (24,26)	24 (23, 26)	<0.001
Degree pursued, n (%)				0.518
Master	1008 (71.5)	535 (72.3)	473 (70.6)	
Doctoral	402 (28.5)	205 (27.7)	197 (29.4)	
Relationship status, n (%)				0.785
Married Partnered Single	87 (6.2) 559 (39.6) 764 (54.2)	51 (6.9) 393 (53.1) 296 (40.0)	36 (5.4) 371 (55.4) 263 (39.2)	
Monthly income (CNY), n (%)				0.002
≤615 616–1,310 1,311–2,086 2,087–3,270 3,271–6,366 >6,367	438 (31.1) 344 (24.4) 341 (24.2) 170 (12.1) 81 (5.7) 36 (2.6)	259 (35.0) 166 (22.1) 187 (25.3) 81 (11.0) 35 (4.7) 14 (1.9)	179 (26.7) 180 (26.9) 154 (23.0) 89 (13.3) 46 (6.9) 22 (3.2)	

**^a^**Pearson’s Chi-squared test; Wilcoxon rank sum test

### Thoughts about dropping out and distress among medical and non-medical postgraduates

The study identified the following prevalence of conditions: depression symptoms (36.2%), anxiety symptoms (27%), somatic symptoms (38.4%), severe stress (40.7%), daytime sleepiness (53.2%), alcohol dependence (14.3%), high level of fatigue (38.9%), low QOL (22.5%), and dropout intention (46.4%). Of the 740 medical postgraduates, 432 (58.4%) had, at some time, thought about dropping out, while only 324 (48.4%) non-medical postgraduates had ever thought about dropping out (p < 0.001). In contrast, medical postgraduates had better overall mental wellbeing than non-medical postgraduates, indicated by a lower prevalence of depression symptoms (33.8% vs. 39.0%, p < 0.001), anxiety symptoms (22.2% vs. 32.4%, p < 0.001), and somatic symptoms (34.7% vs. 42.4%, p = 0.004) among medical students. There were no significant differences in daytime sleepiness (55.0% vs. 51.2%, p = 0.165), high level of fatigue (38.6% vs. 43.0%, p = 0.154), alcohol use (13.4% vs. 15.2%, p = 0.323), or low QOL (21.2% vs. 23.9%, p = 0.173) between the two groups (Table 2).

**Table 2.** Dropout intention and distress among medical postgraduates and non-medical postgraduates

Item	ALL participants (N = 1,410)	Medical postgraduates, (N = 740)	Non-medical postgraduates, (N = 670)	P-value^a^
Thought of dropout, n (%)	654 (46.4)	432 (58.4)	324 (48.4)	<0.001
PHQ9 score, median (IQR)	8 (5,12)	7 (5,11)	8 (6, 12)	0.003
GAD7 score, median (IQR)	6 (4, 10)	6 (3, 9)	7 (4, 11)	<0.001
PHQ15 score, median (IQR)	7 (4, 12)	7 (4, 11)	8 (5, 12)	<0.001
AUDIT-C score, median (IQR)	1 (0, 2)	0 (0, 2)	1 (0, 2)	0.042
ESS score, median (IQR)	11 (8, 14)	11 (8, 14)	11 (8, 14)	0.11
PSS score, median (IQR)	18 (14, 22)	18 (14,22)	18 (15, 23)	0.068
Depression symptoms, n (%)	511(36.2)	250 (33.8)	261 (39.0)	<0.001
Anxiety symptoms, n (%)	381 (27.0)	164 (22.2)	217 (32.4)	<0.001
High perceived stress, n (%)	574 (40.7)	286 (38.6)	288 (43.0)	0.104
Somatic symptoms, n (%)	869 (61.6)	483 (65.3)	386 (57.6)	0.004
Excessive daytime sleepiness, n (%)	750 (53.2)	407 (55.00)	343 (51.2)	0.165
Alcohol abuse/dependence, n (%)	201 (14.3)	99 (13.4)	102 (15.2)	0.323
Fatigue				
Median (IQR)	5 (3,7)	5 (3,7)	5 (3,7)	0.037
High level of fatigue, n (%)	549 (38.9)	276 (37.3)	273 (40.7)	0.154
Quality				
Median (IQR)	6 (5,7)	6 (5,7)	6 (5,7)	0.009
Low level of quality, n (%)	317 (22.5)	157 (21.2)	160 (23.9)	0.173

^a^Pearson’s Chi-squared test; Wilcoxon rank sum test

### Regret, satisfaction, experience of violence, and burnout among medical postgraduates

Over half of the medical postgraduates (396, 53.5%) suffered from burnout, and 450 (60.8%) of them had regretted studying medicine within the last year. Of the 450 respondents who reported their regrets at studying medicine, 362 (80.4%) complained about the long medical training, and 340 (75.5%) were dissatisfied with being overworked. Other reasons for their regrets about studying medicine included low income (306, 68%), intense competition (294, 65.3%), and poor doctor-patient relationships (234, 52%). Over 40% (321, 43.4%) of medical postgraduates were dissatisfied with the medical environment, and over half of this group (384, 51.9%) had experienced violence from patients or their families. See supplementary eTable 1 for more details.

### Medical postgraduates are at higher risk of dropout intention

To further investigate dropout intention in medical postgraduates, we conducted a logistic regression analysis; this analysis revealed that, after full adjustment, medical postgraduates were nearly twice as likely to report turnover intention than non-medical postgraduates. Depression symptoms (OR: 1.92, 95%CI: 1.43–2.58, p < 0.001), high perceived stress (OR: 2.34, 95%CI: 1.78–3.08, p < 0.001), and somatic symptoms (OR: 1.47, 95%CI: 1.12–1.94, p = 0.005) were independently associated with dropout thoughts among all participants. More details are presented in eTable 2.

### Bivariate analysis of dropout intention among medical postgraduates

We then evaluated possible risk factors associated with dropout intention among medical postgraduates, including demographic characteristics, depression symptoms, anxiety symptoms, somatic symptoms, alcohol abuse/dependence, high perceived stress, daytime sleepiness, burnout, QOL, fatigue, experience of violence, and perception of healthcare environment and medical training (Table 3). However, no association between demographic characteristics and dropout thoughts was observed. Thoughts about dropping out were more common among medical postgraduates with depression symptoms (p < 0.001), anxiety symptoms (p < 0.001), daytime sleepiness (p < 0.001), high perceived stress (p < 0.001), high level of fatigue (p = 0.005), low QOL (p < 0.001), burnout (p < 0.001), somatic symptoms (p < 0.001), career choice regret (p < 0.001), dissatisfaction with the medical environment (p < 0.001), and experience of violence (p < 0.001). No significant association was found between alcohol abuse/dependence and thoughts of dropping out among medical postgraduates (p = 0.066).

**Table 3.** Possible factors associated with dropout intention among medical postgraduates

Variable	Without dropout thoughts (N = 308)	With dropout thoughts (N = 432)	p-value^a^
Age, median (IQR)	25 (24, 26)	25 (24, 26)	0.7
Gender, n (%)			0.14
Male	83 (27)	96 (22)	
Female	225 (73)	336 (78)	
Degree pursued, n (%)			1
Master Doctoral	223 (72) 85 (28)	312 (72) 120 (28)	
Monthly income (CNY), n (%)			0.9
≤615	102 (33)	157 (36)	
616–1,310	71 (23)	93 (22)	
1,311–2,086	76 (25)	111 (26)	
v2,087–3,270	36 (12)	45 (10)	
3,271–6,366	17 (5.5)	18 (4.2)	
>6367	6 (1.9)	8 (1.9)	
Career choice regrets, n (%)	106 (34)	344 (80)	<0.001
Violence experience, n (%)	134 (44.6)	248 (57.5)	0.010
Dissatisfaction, n (%)	97 (31.2)	224 (52%)	<0.001
PHQ9 score, median (IQR)	6 (4, 8)	9 (6, 13)	<0.001
GAD7 score, median (IQR)	4 (2, 7)	7 (4, 10)	<0.001
PHQ15 score, median (IQR)	5 (2, 9)	8 (5, 13)	<0.001
ESS score, median (IQR)	10 (7, 13)	12 (8, 15)	<0.001
PSS score, median (IQR)	16 (12, 19)	20 (16, 24)	<0.001
AUDIT3C score, median (IQR)	0 (0, 2)	1 (0, 2)	0.032
Somatic symptoms, n (%)	67 (22)	190 (44)	<0.001
Depression symptoms, n (%)	59 (19)	191 (44)	<0.001
Anxiety symptoms, n (%)	43 (14)	121 (28)	<0.001
Alcohol abuse/dependence, n (%)	34 (11)	65 (15)	0.11
Excessive daytime sleepiness, n (%)	147 (48)	260 (60)	<0.001
High perceived stress, n (%)	68 (22)	218 (50)	<0.001
Fatigue score, median (IQR)	6 (4, 7)	5(3, 7)	0.029
Quality score, median (IQR)	7 (5, 8)	6 (4, 7)	<0.001
High level of fatigue, n (%)	97 (31)	179 (41)	0.006
Low quality of life, n (%)	34 (11)	123 (28)	<0.001
Burnout, n (%)	109 (31.7)	235 (68.3)	<0.001

^a^Wilcoxon rank sum test; Pearson’s Chi-squared test

### Multivariate logistic regression analysis of dropout intention among medical postgraduates

Medical postgraduates showed a lower prevalence of depression, anxiety, and somatic symptoms but a higher risk of dropout, suggesting that experience of violence, perception of the healthcare environment, and medical training might be important factors. Therefore, we performed multivariate stepwise logistic regression (Table 4) to assess the association of violence experience, dissatisfaction, career choice regret, and thoughts about dropping out after adjusting demographic characteristics, QOL, fatigue, and mental health problems (i.e., depression symptoms, anxiety symptoms, somatic symptoms, alcohol abuse/dependence, high perceived stress, burnout, and daytime sleepiness). Dissatisfaction with the healthcare environment (OR: 1.65, 95%CI: 1.17–2.34, p = 0.005) and regrets over choosing a medical career (OR: 6.23, 95%CI: 4.42–8.78, p < 0.001) remained independently associated with thoughts about dropping out. After full adjustment, high perceived stress (OR: 2.74, 95%CI: 1.90–3.94, p < 0.001) was the only mental distress that remained significantly associated with dropout thoughts.

**Table 4.** Logistic regression analysis of factors associated with thoughts of dropout among medical postgraduates

Response	Independent predictor ^a^	Odds ratio (95%CI)	P-value
Thoughts of dropout	Dissatisfaction	1.65 (1.17–2.34)	0.005
	Profession choice regret	6.23 (4.42–8.78)	<0.001
	High perceived pressure	2.74 (1.91–3.94)	<0.001

^a^Variables included demographic characteristics (gender, age, degree pursued, relationship status, and income), quality of life, fatigue, experience of violence, dissatisfaction with healthcare environment, profession choice regrets, and mental distress (daytime sleepiness, acute stress, depression symptoms, anxiety symptoms, somatic symptoms, and burnout)

### The relationship between dissatisfaction, career choice regret, mental distress, and thoughts about dropping out

After adjusting for demographic characteristics, dissatisfaction was found to be independently associated with depression symptoms (OR: 1.93, 95%CI: 1.40–2.65, p < 0.001), anxiety symptoms (OR: 2.14, 95%CI: 1.49–3.07, p < 0.001), high perceived stress (OR: 1.79, 95%CI: 1.70–2.37, p = 0.001), burnout (OR: 2.04, 95%CI: 1.50–2.78, p < 0.001), and somatic symptoms (OR: 2.12, 95%CI: 1.50–2.98, p < 0.001). No association was found between daytime sleepiness, alcohol dependence/abuse, and dissatisfaction.

## Discussion

The present study investigated several mental distress symptoms (i.e., depression symptoms, anxiety symptoms, somatic symptoms, alcohol abuse/dependence, burnout, high perceived stress, and daytime sleepiness) and thoughts about dropping out and their relationship among medical and non-medical postgraduates. The study also explored the risk factors for turnover intention among medical postgraduates. To our knowledge, this study is the first to provide direct evidence that Chinese medical postgraduates are more prone to dropout than non-medical postgraduates. Our study also identified the important role played by the perception of the healthcare environment in the field of mental health and the risk of dropout. Based on the above findings, timely mental health monitoring and intervention for postgraduates and policy changes are called for to reduce the loss of medical postgraduates.

Our survey demonstrated that mental health distress remained common among medical and non-medical postgraduates, even when the COVID-19 situation was improving. In this study, 36.2% of postgraduates reported moderate or higher levels of depression symptoms, which was more severe than before the COVID-19 outbreak (27%) [46] and during the pandemic (34%) in China [47]. Our results were similar to those of surveys carried out among the general population during periods without lockdowns in China [48–53], while higher than the results obtained from healthcare workers in Singapore one year after the outbreak [54]. In addition, the prevalence of depressive symptoms and anxiety symptoms (36.2% and 27.0%, respectively) is higher than that among the general Chinese population (22.6% vs. 3.5% for depression symptoms and 21.4% vs. 7.1% for anxiety symptoms) during similar periods with the use of the same measurements and cutoff points [55–58]. This suggests that postgraduates are at a higher risk of developing depression and anxiety symptoms. Previous studies have demonstrated that prolonged staying at home, academic pressure, especially delays in graduation, excessive exposure to information about the pandemic, unstable income, low level of social support, and anxiety about employment led to distress among college students [59,60]. The high rates of depression and anxiety symptoms after the peak of COVID-19 might reflect the continuation of the psychological harm brought about by the pandemic, as the academic pressure persisted after they returned to campus. From this perspective, reducing academic and employment stress might be helpful.

Previous studies comparing mental health between medical students and their non-medical counterparts have yielded conflicting results. Several studies prior to the outbreak of COVID-19 found that medical students were at a higher risk of psychological distress than non-medical students [61–64]. However, many studies reported a similar or even lower prevalence of anxiety symptoms and depression symptoms in medical students. This result was supported by a meta-analysis involving 12 studies, which showed that only one of the studies demonstrated a higher prevalence of depression symptoms among medical students, when compared with non-medical students [65]. Moreover, after the outbreak of COVID-19, 11 out of 14 studies reported that medical students had better or similar mental health status in China, Poland, France, Saudi Arabia, and Turkey, when compared with non-medical students [49,66–75], which was in line with our study. Only three studies showed a significantly higher level of depression symptoms, anxiety symptoms, and stress among medical students [63,76,77]. Taken together, the above findings might suggest that medical students were not at a higher risk of mental distress compared with their peers in non-medical majors. Moreover, it was indicated that COVID-19 might have varying degrees of impact on the mental health status of medical and non-medical students, the reason for which might be explained as follows. First, a growing body of evidence showed that public education about COVID-19 might help protect people from anxiety and depression symptoms, and medical students are equipped with a better knowledge of COVID-19 [78–81]. Second, medical students demonstrated a positive coping style and stronger psychological resilience [66,82–84], which was a protective factor against mental distress [85]. Third, more engagement in physical activity might be another reason for the better mental well-being of medical students; this was supported by some previous studies, which demonstrated that physical activity was negatively associated with mental distress [86] and that medical students reported a higher level of physical activity during the lockdown [87]. This finding was also supported by another cross-sectional study, in which medical students engaged in less physical activity and showed a higher level of anxiety compared to students engaging in high levels of physical activity [63]. Based on our findings, timely psychological counseling and intervention, especially for non-medical students, are required. Further studies are needed to investigate the dynastic changes in mental health status in medical and non-medical students.

Furthermore, the prevalence of dropout intention was significantly higher among medical postgraduates (58.4%) than non-medical postgraduates (48.4%), despite the better overall mental health status among medical students. After full adjustment, medical postgraduates were nearly twice as likely to report turnover intention than non-medical postgraduates, suggesting that there might be other risk factors associated with their dropout intention in addition to mental health status. Given the dual role of medical postgraduates, that is, they are both physicians and students, we hypothesized that perception of the healthcare environment might play a role in their turnover intention and a logistic regression model was used to investigate.

The logistic regression model revealed the importance of attitudes toward the healthcare environment and medical training on depression symptoms, anxiety symptoms, high stress, burnout, somatic symptoms, and dropout. Medical postgraduates with regrets about studying medicine are five times more likely to consider leaving the medical profession. Based on our findings, the reasons include the long medical training period, overwork, low income, intense competition, poor doctor-patient relationships, workplace violence, and unrealistic expectations of patients. Despite the differences in training modes and healthcare environments, several studies are comparable to ours [88–91]. In line with previous studies, we found that depression, burnout, and high perceived stress were major manifestations of medical students’ distress, which led to them leaving the profession [91]; our study also identified the impact of a negative perception of the healthcare environment on the students’ distress. Among healthcare providers worldwide, dissatisfaction with income and overwork is prevalent and contributes to loss from the workforce in this field [89,90]. Studies on Chinese medical undergraduates and physicians identified similar reasons for quitting medical practice or quitting medical studies [16,35,36,92,93]. This may suggest that these risk factors affect doctors’ medical careers at a very early stage, and the impact persists, which may lead to their eventual loss from the medical workforce.

In China, long medical training periods, long working hours, and low income are considered to be the ‘sacrifices’ made by physicians, directly leading to the loss of medical undergraduates and physicians [36,92,93]. Most medical students pursuing a master’s degree in China must undergo at least eight years of training, which is typically one or two years longer than non-medical students [94]. The double-track training system and reform in medical education have added to the burden on medical postgraduates, as the new system requires postgraduate students to complete scientific research training alongside their residency training. According to the National Bureau of Statistics, physicians’ average working hours in China are 50.90 and 49.79 hours for males and females, respectively, compared to 46.3 hours for non-medical workers [95,96]. The long working hours, especially the night and weekend duties, are strongly related to their intention to leave the medical profession [15,93]. Moreover, the amount of overtime worked by physicians is often underestimated and unpaid. Many Chinese physicians complain about their excessive workloads and disproportionately low incomes [15,97]. According to a recent study, the incomes of physicians in public hospitals are much lower than those in the Economic Co-operation and Development countries and do not reflect the true human capital value of physicians [98]. Unfortunately, the situation has not been alleviated by the medical reforms of the past five years. Two cross-sectional surveys investigated the turnover intention and satisfaction of physicians in 2016 and 2020 and reported the same reasons for physicians leaving the profession as mentioned above [16,99,100].

Poor doctor-patient relationships, including violence and declined physician reputation, also play their part in the risk of doctors dropping out. A survey in 2018 revealed that 66% of physicians in China experienced conflict with patients, with more than 30% having experienced violence [101]. In the present study, half of the medical postgraduates experienced verbal or physical violence, indicating the vulnerability of medical postgraduates, who often work as primary doctors in China; their experience of violence was found to be strongly associated with turnover intention. Apart from violence, the declined professional reputation of doctors was another risk factor for burnout and turnover intention [102]. Our findings regarding this issue are similar to those of another study reporting that undergraduates are 1.26 times more likely to give up their medical career when they believe that ‘doctor is not an occupation with a high social status and reputation’ [36]. Therefore, government support, positive media guidance, and communication skills are urgently needed for practicing doctors. Screening and monitoring of psychological distress among medical students are also needed. In the future, more measures can be taken to address the issue of loss of workforce in the medical field, such as providing a shorter training period for doctors, allowing flexible working hours with fewer overnight and weekend duties, improving income levels, especially providing overtime pay, as well as raising the profile of medical professionals.

## Limitations

Our study had several limitations. First, a cross-sectional survey precluded us from identifying a causal relationship. Second, the use of the convenience sampling method may have induced selection bias, and the limited sample size might not have been representative enough of the huge number of postgraduate students in China. Third, there might have been a respondent bias, as individuals with psychological distress were more willing to participate in this survey because we provided a report of their mental health status and psychological counseling as needed upon completion of this questionnaire. However, the prevalence of depression symptoms, anxiety symptoms, excessive daytime sleepiness, and dropout intention was similar to previous studies [36,47,103,104], suggesting the effects of such bias on our results was likely to be insignificant. Finally, we used a self-rating scale rather than a standardized diagnostic tool to determine the incidence of mental distress, which might have reduced the accuracy of the results. Thus, a prospective longitudinal study with a larger sample size of postgraduates is warranted.

## Conclusion

In conclusion, our study demonstrated a high prevalence of depression symptoms, anxiety symptoms, somatic symptoms, alcohol abuse/dependence, high stress, and excessive daytime sleepiness in postgraduates, indicating the need for a full screening of those with mental distress among college students. To our knowledge, this is the first study to reveal that the relatively high level of dropout intention among Chinese medical postgraduates is associated with stress, dissatisfaction with the medical environment, and career choice regrets. Therefore, there is an urgent need for improvement in health policies to make medical postgraduates stay in the profession.

## Supplementary Material

Supplemental Material
