# Supplementary material for: High prevalence and risk factors of dropout intention among Chinese medical postgraduates
Source: Med Educ Online. 2022 Mar 31;27(1):2058866. doi: 10.1080/10872981.2022.2058866 (PMC8979499; doi:10.1080/10872981.2022.2058866)
Supplement: Supplemental Material [file ZMEO_A_2058866_SM7748.docx]

Supplementary materials for “High Prevalence and Risk Factors of Dropout Intention among Chinese Medical Postgraduates”

**eTable1.** Regret, satisfaction, violence experience, and burnout among medical postgraduates

**eTable2.** Logistic Regression Modeling of Factors Associated with Thoughts of Dropout among Postgraduates

**eTable1 Regret, Dissatisfaction, Violence experience, and Burnout among medical postgraduates**

| **Item** | **Medical postgraduates, (n=740)**  **n (%)** |
| --- | --- |
| Burnout |  |
| Emotional exhaustion | 328(44.3) |
| Personal depersonalization | 121(16.4) |
| Satisfaction with the medical environment |  |
| Dissatisfied | 321(43.4) |
| Satisfied or neutral | 419(56.6) |
| Violence from patient  Not experienced  Experience verbal violence  Experience physical violence  Experience physical and verbal violence  Career choice regret  Yes  No  Reasons for regret  Long study period  Overwork  Low income  Intensive competition  Poor doctor-patient relationship  Violence  High expectation of patients  Other causes | 356(48.1)  339(45.8)  7(0.9)  38(5.1)  450(60.8)  290(30.2)  362(80.4)  340(75.5)  306(68.0)  294(65.3)  234(52.0)  226(50.2)  174(38.6)  89(19.8) |

**eTable2 Logistic Regression Modeling of Factors Associated with Thoughts of Dropout among Postgraduates**

| **Response** | **Independent**  **predictor ^a^** | **Odds ratio (95% CI)** | **P-value** |
| --- | --- | --- | --- |
| Thoughts of dropout | Being medical postgraduate ^b^ | 1.76(1.40-2.21) | <0.001 |
|  | Moderate or severe depression | 1.92(1.43-2.58) | <0.001 |
|  | High pressure | 2.34(1.78-3.08) | <0.001 |
|  | Moderate or severe somatization | 1.47(1.12-1.94) | 0.005 |

^a^  Variables include demographic characteristics (gender, age, major, degree applied for, income, relationship status, income), quality of life, fatigue, and mental distress (Alcohol dependence, daytime sleepiness, acute stress, moderate or severe depression, anxiety, somatization)

^b^  Compared with non-medical postgraduates.
